# Presentation and surgery outcomes in elderly with pheocromocytoma: a comparative analysis with young patients

**DOI:** 10.1590/S1677-5538.IBJU.2015.0503

**Published:** 2016

**Authors:** Victor Srougi, Jose L. Chambo, Fabio Y. Tanno, Iracy S. Soares, Madson Q. Almeida, Maria A. A. Pereira, Miguel Srougi, Maria C. Fragoso

**Affiliations:** 1 Divisão de Urologia do Hospital das Clínicas da Universidade de São Paulo, Faculdade de Medicina, São Paulo, Brasil; 2 Divisão de Anestesiologia do Hospital das Clínicas da Universidade de São Paulo, Faculdade de Medicina, São Paulo, Brasil; 3 Divisão de Endocrinologia do Hospital das Clínicas da Universidade de São Paulo, Faculdade de Medicina, São Paulo, Brasil

**Keywords:** Pheochromocytoma, Adrenalectomy, Surgical Procedures, Operative

## Abstract

**Purpose:**

To evaluate the presentation and early surgical outcomes of elderly patients undergoing adrenalectomy for phaeochromocytoma.

**Patients and Methods:**

A retrospective search was performed of our adrenal disorders database for patients who underwent surgery for phaeochromocytoma or paraganglioma between 2009 and 2014. Patients >60 years old were classified as elderly. The clinical manifestations, intraoperative course, and early postoperative outcomes of elderly patients were compared to those of younger individuals (<60 years old).

**Results:**

The mean (±standard deviation) age in the older (n=10) and younger (n=36) groups was 69.6±5.3 years and 34.0±12.9 years. Germ-line mutations were more common in younger patients (50.0% versus 0%; p=0.004), whereas incidental lesions were more common in the elderly (40.0% versus 5.3%; p=0.003). In both groups, surgery was most commonly performed by videolaparoscopy (90% in the elderly and 82% in the younger group), with similar intraoperative anesthetic and surgical outcomes. Postoperatively, the older group more commonly received vasoactive drugs (60.0% versus 10.5%; p<0.001) and had a longer intensive care unit stay (3.1±2.8 versus 1.4±1.0 days; p=0.014), more clinical complications (60% versus 18.9%; p=0.01), and longer hospital stay (10.2±8.4 versus 5.7±4.9 days; p=0.028).

**Conclusions:**

Although all patients received the same preoperative preparation, the elderly group exhibited a slower and more complicated recovery after adrenalectomy. Meticulous perioperative care should be used in the elderly when treating phaeochromocytoma; nevertheless, adrenalectomy is a relatively safe procedure in this patient population.

## INTRODUCTION

Phaeochromocytoma is a neuroendocrine tumor with a prevalence ranging from 0.05% to 0.1% (1, 2). Phaeochromocytomas are located in the adrenal cortex in 85% of cases (2). The main clinical manifestation of phaeochromocytomas is arterial hypertension, which is the result of uncontrolled production of catecholamines by the tumor. It is estimated that 0.1% to 0.6% of all patients with hypertension have an underlying phaeochromocytoma (2, 3). Moreover, catecholamine-producing neoplasms can lead to hypertensive emergencies with life-threatening consequences; thus, this tumor has major clinical importance (2, 4).

Curative treatment for phaeochromocytoma requires surgical removal of the tumor, which can be challenging because of the high likelihood of hemodynamic instability during the procedure and substantial blood pressure fluctuations during the recovery period (5). Fortunately, the development of preoperative strategies for strict blood pressure control has led to an important reduction in mortality rates. Forty years ago, the perioperative mortality ranged from 30% to 45% (5), whereas in recent series, perioperative mortality decreased to 0% to 2.9% (1, 3). Surgery-related complications have also declined, but they are still frequent, occurring in one of every five patients who undergo surgery (4).

Phaeochromocytomas are frequently associated with familial syndromes; in these instances, the tumors are usually diagnosed in young adults. However, phaeochromocytomas also affect children and the elderly, with sporadic cases being more common in older patients (2). Furthermore, with recent increases in life expectancy and improvements in imaging techniques, there has been an increase in the number of incidental diagnoses of phaeochromocytoma in older individuals undergoing periodic routine check-ups (6).

As an increasing number of the elderly are being considered for surgical removal of phaeochromocytomas, concerns have arisen regarding the perioperative management of older individuals undergoing this surgery. The elderly are physically more fragile than younger adults because of their attenuated systemic response to surgical stress and the frequent presence of comorbidities. Thus, surgical outcomes in this population may be worse than in younger patients (7, 8). We therefore performed the present study to evaluate the presentation and surgical outcomes of elderly individuals with phaeochromocytoma.

## PATIENTS AND METHODS

After ethics board approval, a retrospective review of the adrenal disorders database at our institution was performed, selecting patients with phaeochromocytoma and paraganglioma who underwent adrenalectomy or adrenal tumor resection between January 2009 and January 2014. The diagnosis was confirmed by histopathological analysis. Forty-nine patients were identified, three of whom were excluded because of incomplete data. Two individuals with a metachronous contralateral phaeochromocytoma underwent two operations, resulting in a total of 48 procedures. The patients were divided into two groups according to their age at surgical intervention. Individuals older than 60 years were classified as elderly, in accordance with World Health Organization (WHO) recommendations (9). These recommendations state that the age cutoff to define elderly is established by the local government, with a minimum age of 60 years.

Preoperative data were reviewed, with comorbidities classified according to the Charlson scoring system (10). The clinical presentation at tumor diagnosis was categorized as typical manifestation, atypical manifestation, incidental finding, or familial syndrome follow-up. Typical symptoms included the presence of palpitations, headaches, sweating, syncope, tremors, weight loss, and hypertensive crisis. The presence of any other symptom was considered an atypical manifestation. Incidental diagnosis was defined as the presence of tumor in an asymptomatic patient who had an unexpected adrenal mass detected on imaging examination, in the absence of a familial syndrome.

According to our institution's clinical guidelines, all patients had a thorough preoperative evaluation and only underwent surgery after their comorbidities were strictly controlled. Selective alpha-receptor blockers were administered preoperatively in all patients. Either prazosin and doxazosin were initiated at least 15 days before surgery, with the doses adjusted to achieve a blood pressure <135/85mmHg. In patients with a heart rate >100 beats/min, beta-blockers were also administered. Two days before surgery, the patients began a liquid diet, and on the evening before surgery, they received 10mg bisacodyl orally for bowel cleaning.

All surgical procedures were performed by the same experienced surgeon, supervising a urology resident. The choice of surgical modality was based on the number of previous abdominal surgeries and whether the tumor was suspected to be malignant. Transperitoneal videolaparoscopy was the first choice whenever possible; it was initiated in 40 cases, although three required conversion to an open technique. The other eight adrenalectomies were accomplished by the open approach. In patients with bilateral phaeochromocytomas, total adrenalectomy was performed first on the side with the higher tumor volume and partial adrenalectomy was performed when possible in the contralateral gland. All patients were anesthetized by the same skilled professional, who was supervising an anesthesia resident. The diagnosis of phaeochromocytoma was confirmed after analysis of the surgical specimen. Intraoperative events, including surgical and anesthesia data, were reviewed. Clinical complications, stratified by the Clavien score (11), and early postoperative events were also evaluated.

Data are presented as mean±standard deviation unless otherwise indicated. Statistical analysis was performed with the SPSS^®^ program, with the student-test or Kruskal-Wallis test being used for continuous variables. Chi-squared or Fisher tests were used to compare categorized variables. Values were considered significant if the p value was <0.05.

## RESULTS

Among the 46 patients evaluated, 10 were included in the elderly group (five males and five females) and 36 in the young adult group (nine males and 27 females). The respective mean ages of the two groups were 69.6±5.3 years and 34.0±12.9 years. Two individuals in the younger group with metachronous phaeochromocytoma underwent two operations, for a total of 38 surgeries in this group. The mean Charlson score was 3.6±1.3 in the older group and 0.89±1.0 in the younger group (p<0.0001). No genetic alterations were observed in the elderly group, whereas in the younger group, 18 individuals (50.0%) had germ-line mutations (p=0.004). Incidental diagnosis was noted in 40.0% of patients in the older group but in only 5.3% of patients in the younger group (p=0.003). Data regarding the diagnoses at presentation are shown in Table-1.

**Table 1:** Pre-operative characteristics.

		Elderly	Young	P
No.		10	36	
Average age		69.6 ± 5.3	34.0 ± 12.9	
Male: Female		5:5	9:27	0.128
Mean Charlson		3.6 ± 1.3	0.89 ± 1.0	< 0.0001
Germ-line mutation {%)		0	18(50%)	0.004
**Diagnosis** (%)				
	Incidental	4 (40%)	2 (5.3%)	0.003
	Typical symptoms	3 (30%)	16(42.1%)	0.486
	Atypical symptoms	3 (30%)	7(18.4%)	0.422
	Investigation / follow-up on familial syndrome	0	13(34.2%)	0.030
Long-term hypertension (%)		8 (80%)	16(43.2%)	0.039
Anti-hypertensive drugs (number)		1.7 ± 1.2	0.6 ± 1.0	0.008
**Image evaluation**				
	Bilateral (%)	0	11/36(30.6%)	0.045
	Average lesion size (cm)	5.31 ± 2.4	4.2 ± 2.6	0.219
	Positive cyntigraphy-MIBG (%)	9/10(90%)	20/24 (83.3%)	0.616

Long-term hypertension was a common finding in both groups, but it was more frequent among the elderly (80.0% versus 43.2%; p=0.039). The number of medications used to control the blood pressure preoperatively was also significantly higher among the elderly (1.7±1.2 versus 0.6±1.0; p=0.008). The mean tumor size was 5.3±2.4cm and 4.2±2.6cm in the older and younger groups, respectively (p=0.219). Metaiodobenzylguanidine-scintigraphy was performed in 34 patients; positive results were found in a similar percentage of older and younger patients (90.0% in the older group and 83.3% in the younger group; p=0.616).

Among the 40 attempted videolaparoscopic adrenalectomies (9 [90%] in the older group and 31 [82%] in the younger group), three were converted to open surgery: one in the older group because of difficulty performing the adrenal dissection, and two in the younger group because of uncontrolled bleeding. The other eight surgeries were performed by the conventional open approach.

Eleven (29%) patients in the younger group had bilateral tumors; six of these were synchronous. In these patients, bilateral adrenalectomy was performed, removing the entire gland on the side with the largest tumor and preserving approximately 1/3 of the adrenal gland on the other side. Among the patients with metachronus tumors who previously underwent total adrenalectomy, three underwent partial adrenalectomy and two underwent total adrenalectomy. There were no bilateral phaeochromocytomas in the older group. Two cases of paraganglioma were diagnosed, one in each group; the open approach was used for the younger group patient and videolaparoscopic surgery was used in the older group patient. There were two cases of malignant tumors, both of which were in the younger group (5.5%).

The early perioperative outcomes are displayed in Table-2. There were no significant differences between groups in any of the evaluated intraoperative parameters. In the postoperative period, the elderly had a higher need for vasopressors (60.0% versus 10.5%; p<0.001), longer intensive care unit (ICU) stay (3.1±2.8 days versus 1.4±1 days; p=0.014), and longer hospital stay (10.2±8.4 days versus 5.7±4.9 days; p=0.028). Postoperative complications occurred in 60% of the older patients but only 18.9% of the younger patients (p=0.01). According to the Clavien classification, there were five minor complications and one major complication (pulmonary thromboembolism requiring ICU admission) in the older group, whereas there were seven minor complications in the younger group (Table-3). There were no deaths in any patient.

**Table 2:** Intra-operative and early post-operative outcomes.

		Elderly	Young	p
**Surgery Modality** (%)				
	VLP	9 (90%)	31 (82%)	
	Open	1 (10%)	7(18%)	
Partial Adrenalectomy		0	9 (23.7%)	
Synchronous Bilateral Adrenalectomy		0	6(16%)	
**Intra-operative outcomes**				
	Surgery conversion {%)	1/9(11.1%)	2/31 (6.5%)	0.640
	Hypertension / Tachycardia {%)	9 (90%)	32 (84.2%)	0.644
	Vasoactive drugs use {%)	9 (90%)	28 (73.7%)	0.274
	Average Crystalloid volume (mL)	3500 ± 1130	3658 ± 1048	0.696
	Average anesthesia time (min)	261 ± 73	290 ± 73	0.291
	Average surgery time (min)	161 ± 47	168 ± 66	0.722
	Blood transfusion (%)	1 (10%)	8(21.1%)	0.426
**Early post-operative outcomes**				
	ICU alter surgery	8 (80%)	33 (86.8%)	0.585
	Average time on ICU (days)	3.1 ± 2.8	1.4 ± 1	0.014
	Need of vasoactive drugs (%)	6 (60%)	4 (10.5%)	< 0.001
	Clinical complications (%)	6 (60%)	7 (18.9%)	0.010
	Average hospital discharge (days)	10.2 ± 8.4	5.7 ± 4.9	0.028

**Table 3:** Complications according to Clavien classification.

		Elderly	Young	p
Complications		6 (60%)	7(18.9%)	0.010
**Minor complications**				
	Clavien I	2	4	
	Clavien II	3	3	
**Major complications**				
	Clavien III	0	0	
	Clavien IV	1	0	

## DISCUSSION

Considering the complexity of the perioperative management of phaeochromocytomas, we hypothesized that elderly patients would have worse outcomes compared to younger patients. Our results showed that surgery in the elderly was feasible, but recovery was slower and more prone to postoperative complications.

The diagnosis of neoplasms in the elderly has increased in recent years because of the rise in life expectancy and the implementation of routine screening exams (7). Surgeons may be hesitant to perform aggressive treatment in this population because of their higher risk of perioperative complications and death (12, 13). As a consequence of aging, breathing capacity, renal function, and resting cardiac output are reduced. Moreover, baseline global metabolism declines, thereby attenuating the body's physiologic response to stress (7, 8). However, when a catecholamine-producing tumor is discovered, expectant management is not appropriate. Besides the undesirable effects of sustained elevated blood pressure, these tumors can produce hypertensive emergencies, with potentially life-threatening cardiovascular consequences (2). An autopsy study reported that 75% of deaths related to phaeochromocytoma were due to a myocardial infarction or cerebral vascular accident (14), emphasizing the importance of interventional treatment.

Surgery in the elderly requires maximum effort, focusing on strict control of comorbidities and thorough surgery preparation. Associated diseases may impair surgical recovery by increasing complications and raising mortality rates (8, 13). Our findings support these observations, as both the Charlson score (3.5 versus 0.89) and rate of clinical postoperative complications (60% versus 18%) were higher in the elderly. The complication rate noted in our younger patients was similar to rates reported in the literature, which varied from 10% to 21% (4, 15, 16). The elderly also had a six times higher likelihood of receiving vasopressors postoperatively, compared to younger patients. Furthermore, during recovery, older patients had a longer duration of ICU stay and hospitalization. However, intraoperative parameters were similar in the two groups. Bruynzeel et al. (3) also did not find a relationship between age and hemodynamic instability during surgery for phaeochromocytoma. Other previous studies have included elderly patients in their study population, but none have analyzed outcomes stratified by age.

In addition to age and comorbidities, the tumor volume and presence of familial syndromes may also influence surgical outcomes. Younger patients had a 50% rate of germ-line mutations, which are frequently associated with bilateral and malignant phaeochromocytomas (2, 4). Regarding tumor volume, a positive relationship between tumor size and higher catecholamine production has been previously described (3). The increased catecholamine release could produce more intense symptoms and more severe illness. In the present study, there was no difference in tumor size between groups, and the expected relationship between familial syndromes and worse outcomes was not observed.

Regarding preoperative care, several actions should be adopted in the elderly to facilitate a quicker surgical recovery with fewer complications. Although the use of alpha-blockers is indisputable, there is controversy regarding which alpha-blocker medication most effectively optimizes outcomes (2). Although no correlation with age has been reported, short-acting selective alpha-blockers can avoid prolonged postoperative hypotension and are thereby generally preferred for the elderly (16). Furthermore, preoperative bowel preparation should be judicious in the elderly, to prevent hydric and electrolyte disturbances and to minimize the risks of colonic bacterial translocation (17). To reduce the risk of postoperative pulmonary embolism, anti-coagulation regimens must be introduced early, as difficulty with postoperative ambulation is anticipated. Intense pulmonary rehabilitation should be instituted to reduce the risk of other postoperative pulmonary complications.

This study is subject to the drawbacks of its retrospective design, small sample size and absence of matched paired comparison. Moreover, as our department is a quaternary referral service, many patients returned to their original health care center and were lost to long-term follow-up at our institution. However, this is the first series specifically addressing the feasibility of phaeochromocytoma resection in the elderly population. Furthermore, all patients were treated by the same surgeon who had performed more than 300 adrenalectomies in the preceding 10 years.

In conclusion, our elderly population had a slower recovery and more complications after resection of phaeochromocytoma, when compared to young adults. However, most complications were minor, surgery was equally feasible and did not result in mortality in this vulnerable group of patients. Meticulous preparation for surgery is crucial among the elderly and particular attention should be focused on maintaining balance between the adrenal disease and comorbidities.

## References

[1] Lenders JW, Eisenhofer G, Mannelli M, Pacak K (2005). Phaeochromocytoma. Lancet.

[2] Minno AM, Bennett WA, kvale WF (1954). Pheochromocytoma; a study of 15 cases diagnosed at autopsy. N Engl J Med.

[3] Bruynzeel H, Feelders RA, Groenland TH, van den Meiracker AH, van Eijck CH, Lange JF (2010). Risk Factors for Hemodynamic Instability during Surgery for Pheochromocytoma. J Clin Endocrinol Metab.

[4] Williams DT, Dann S, Wheeler MH (2003). Phaeochromocytoma-views on current management. Eur J Surg Oncol.

[5] Hull CJ (1986). Phaeochromocytoma. Diagnosis, preoperative preparation and anaesthetic management. Br J Anaesth.

[6] Kalache A, Keller I, Evans JG, Williams TF, Beattie BL, Michel JP, Wilcock GK (2000). Population ageing in developing countries: demographic aspects. Oxford Textbook of Geriatric Medicine.

[7] Zenilman ME (1998). Surgery in the elderly. Curr Probl Surg.

[8] Ramesh HS, Pope D, Gennari R, Audisio RA (2005). Optimising surgical management of elderly cancer patients. World J Surg Oncol.

[9] World Health Organization Health statistics and information systems - Definition of an older or elderly person.

[10] Charlson ME, Pompei P, Ales KL, MacKenzie CR (1987). A new method of classifying prognostic comorbidity in longitudinal studies: development and validation. J Chronic Dis.

[11] Clavien PA, Barkun J, de Oliveira ML, Vauthey JN, Dindo D, Schulick RD (2009). The Clavien-Dindo classification of surgical complications: five-year experience. Ann Surg.

[12] Damhuis RA, Meurs CJ, Meijer WS (2005). Postoperative mortality after cancer surgery in octogenarians and nonagenarians: results from a series of 5,390 patients. World J Surg Oncol.

[13] New Classification of Physical status (1963). American Society of Anaesthesiologists. Anaesthesiology.

[14] Sutton MG, Sheps SG, Lie JT (1981). Prevalence of clinically unsuspected pheochromocytoma. Review of a 50-year autopsy series. Mayo Clin Proc.

[15] Kiernan CM, Du L, Chen X, Broome JT, Shi C, Peters MF (2014). Predictors of hemodynamic instability during surgery for pheochromocytoma. Ann Surg Oncol.

[16] Weismann D, Fassnacht M, Weinberger F, Hamelmann W, Diehl S, Lorenz K (2006). Intraoperative haemodynamic stability in patients with phaeochromocytoma––minimally invasive vs conventional open surgery. Clin Endocrinol (Oxf).

[17] Compagna R, Aprea G, De Rosa D, Gentile M, Cestaro G, Vigliotti G (2014). Fast track for elderly patients: is it feasible for colorectal surgery?. Int J Surg.

